# Therapeutic efficacy of β-sitosterol treatment on *Trypanosoma congolense* infection, anemia development, and trans-sialidase (*TconTS1*) gene expression

**DOI:** 10.3389/fmicb.2023.1282257

**Published:** 2023-09-27

**Authors:** Suleiman Aminu, Gloria Dada Chechet, Samia S. Alkhalil, Mansour Sobeh, Rachid Daoud, Mthokozisi B. Simelane, Elewechi Onyike, Mohammed Auwal Ibrahim

**Affiliations:** ^1^Department of Biochemistry, Ahmadu Bello University, Zaria, Nigeria; ^2^Chemical and Biochemical Sciences-Green Processing Engineering, Mohammed VI Polytechnic University, Ben Guerir, Morocco; ^3^African Center of Excellence for Neglected Tropical Diseases and Forensic Biotechnology, Ahmadu Bello University, Zaria, Nigeria; ^4^Department of Clinical Laboratory Sciences, College of Applied Medical Sciences, Shaqra University, Alquwayiyah, Saudi Arabia; ^5^AgroBioSciences Program, College for Sustainable Agriculture and Environmental Science, Mohammed VI Polytechnic University, Ben Guerir, Morocco; ^6^Department of Biochemistry, University of Johannesburgs, Johannesburg, South Africa

**Keywords:** anemia, **β**-sitosterol, organ damage, renal hypertrophy, *Trypanosoma congolense*, trans-sialidase

## Abstract

**Background:**

African animal trypanosomiasis hinders sustainable livestock productivity in sub-Saharan Africa. About 17 million infected cattle are treated with trypanocides annually but most of the drugs are associated with drawbacks, necessitating the search for a promising chemotherapeutic agent.

**Objectives:**

In this study, the effects of β-sitosterol on *Trypanosoma congolense* infection were investigated along with its effect on the trans-sialidase gene expressions.

**Results:**

Oral treatment with β-sitosterol at 15 and 30 mg/kg body weight (BW) for 14 days significantly (*p* < 0.05) reduced parasitemia and ameliorated the parasite-induced anemia. Also, the parasite-induced increase in serum urea level and renal histopathological damage scores in addition to renal hypertrophy was significantly (*p* < 0.05) reverted following treatment with 30 mg/kg BW β-sitosterol. The compound also significantly (*p* < 0.05) down-regulated the expression of *TconTS1* but not *TconTS2*, *TconTS3*, and *TconTS4*. Correlation analysis between free serum sialic acid with the *TconTS1* and *TconTS2* gene variants revealed negative correlations in the β-sitosterol-treated groups although they were non-significant (*p* > 0.05) in the group treated with 15 mg/kg BW β-sitosterol. Similarly, a non-significant negative (*p* > 0.05) correlation between the biomolecule and the *TconTS3* and *TconTS4* gene variants was observed in the β-sitosterol-treated groups while positive correlations were observed in the infected untreated control group.

**Conclusion:**

The observed effect of β-sitosterol on *T. congolense* infection could make the compound a possible template for the design of novel trypanocides.

## 1. Introduction

As the world intensifies its effort toward searching for an ultimate therapy against diseases such as COVID-19 infection (Weiner et al., 2020), African nations are mainly looking inward to tackle the menace of tropical parasitic diseases such as African Trypanosomiasis (Garchitorena et al., 2017). These diseases not only affect human or animal health but also affect the economic well-being of the continent (Sun and Amon, 2018). One of the most important tropical diseases is African Animal Trypanosomiasis (AAT) caused by parasitic protozoan species including *Trypanosoma congolense (T. congolense)*, *Trypanosoma vivax (T. vivax)*, and *Trypanosoma brucei brucei (T. brucei brucei)* that are spread across the 37 sub-Saharan African countries where they affect livestock production (Habeeb et al., 2021).

Among the species mentioned, *T. congolense* has been considered the second most virulent and the most pathogenic species in cattle leading to AAT (Katabazi et al., 2020). After infection, the parasite develops extracellularly and interacts with circulating erythrocytes and vital organs of the infected host thereby causing profound damage at the adhesion sites. Importantly, the interaction imposes systemic oxidative stress that ultimately leads to hemolytic anemia and degenerative changes in the organs (Ibrahim et al., 2016). In fact, the above disease manifestations have been considered as factors resulting in the ultimate death of the infected host whilst anemia has been considered the major and most prominent symptom of the disease (Ibrahim et al., 2016; Saad et al., 2020).

Although multiple pathophysiologic mechanisms have been proposed to be responsible for anemia generation, the involvement of (trans)-sialidase has been reported to be the major etiologic agent (Coustou et al., 2012; Mbaya et al., 2012; Balogun et al., 2014). The *T. congolense* trans-sialidase (TconTS) has been implicated in the hydrolysis and transfer of host sialic acid for sialylation of the parasite surface molecules (Haynes et al., 2015). The transfer process is essential to the parasite as a survival strategy to escape the host defense mechanism since the sialic acid is abundantly found in the parasite Variant Surface Glycoprotein (VSG) coats responsible for antigenic variation (Quintana et al., 2018). Historically, within the African trypanosomes, trans-sialidases were initially found in procyclic *T. brucei brucei* with 38% similarities to *T. cruzi* trans-sialidase with most of the amino acid residues in the active sites conserved (Montagna et al., 2002). Two variants of the enzyme were found in procyclic *T. congolense* with conserved amino acid residues in the active site (Tiralongo et al., 2003; Montagna et al., 2006). However, further investigation revealed the presence of trans-sialidases in the bloodstream form of *T. congolense* (Coustou et al., 2012) where four active variants were recognized namely; *TconTS1*, *TconTS2*, *TconTS3*, and *TconTS4* (Amaya et al., 2004; Gbem et al., 2013). Distinctively, *TconTS1* and *TconTS2* have a high trans-sialidase activity ratio while the *TconTS3* and *TconTS4* exclusively perform sialidase activity. The utilization of these variants to either cleave and/or transfer sialic acid depends on the parasite’s needs at a particular instant. On the other hand, the de-sialylation of the host RBCs leads to erythrophagocytosis and subsequently anemia (Balogun et al., 2014).

With the tremendous negative threats of AAT, exploiting strategies to control the disease is imminent (Samdi et al., 2010). As antigenic variation poses a great challenge in the production of vaccines (Onyilagha and Uzonna, 2019), the available options for disease control primarily centered on vector control or the use of trypanocides (Gimonneau et al., 2018). Since vector control was expensive on a large scale with little success, chemotherapy, therefore, serves as the gold standard approach to manage the disease (Eghianruwa and Oridupa, 2018). Currently, most of the drugs against the disease are limited due to many factors in addition to low therapeutic indices, resistance, and toxicity (Meyer et al., 2016; Assefa and Shibeshi, 2018). For instance, diminazene aceturate as one of the drugs used to treat trypanosomiasis was reported to induce adverse effects on the central nervous system and was associated with several signs of toxicity in the blood and systemic organs, as observed with changes in hematological and biochemical parameters. High doses of the drug eventually lead to mortality (da Silva Oliveira and da Silva, 2022). Moreover, clinical signs such as depression, ataxia, and seizures are associated with diminazene toxicity (Han et al., 2014). Hence, there is a need to develop lead compounds or structural scaffolds that could aid in the discovery of chemotherapeutic agents that could hinder the disease progression.

**Figure 1:**
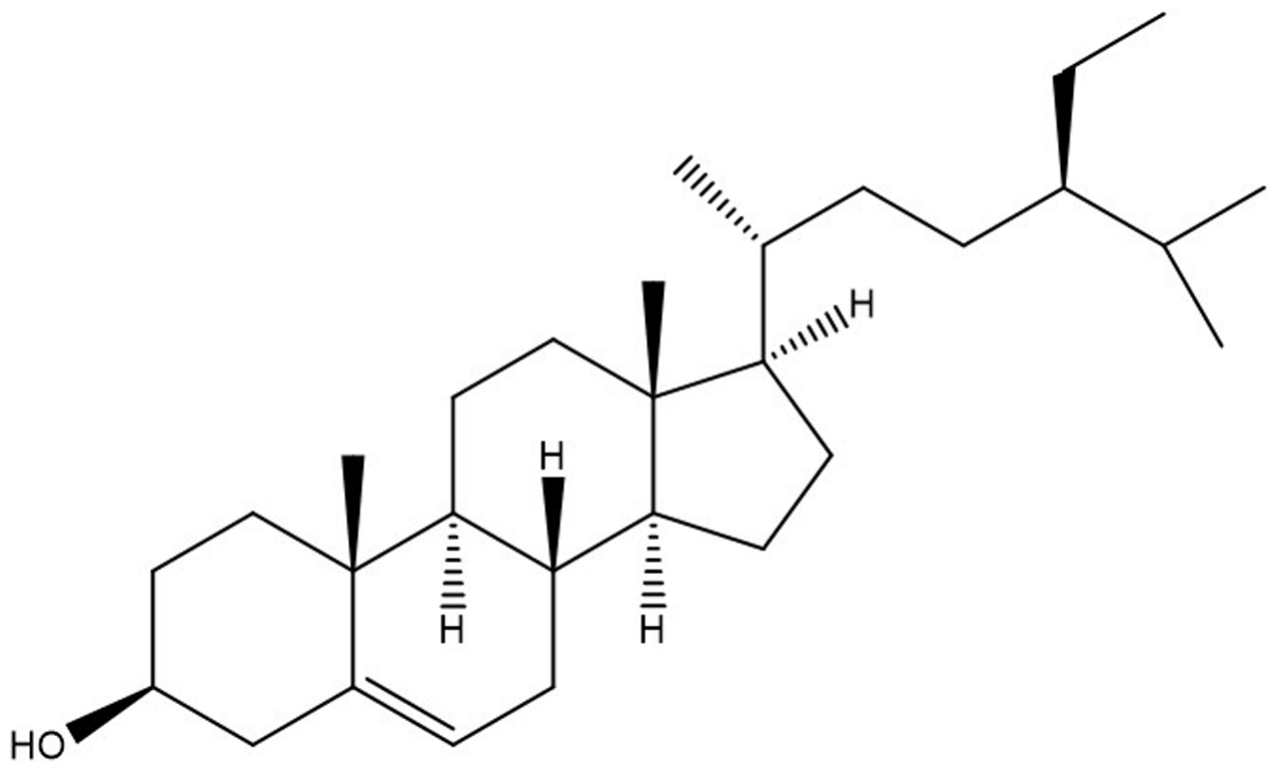
Structure of β-sitosterol.

The propensity of information has confirmed that African medicinal plants possess bioactive compounds with high *in vitro* antitrypanosomal efficacy (Ibrahim et al., 2014). Specifically, some of the compounds were proven to have a positive effect against *T. congolense*-induced pathologies even though complete elimination of the parasite from the bloodstream of infected animals was not achieved (Aminu et al., 2017a,b, 2022; Saad et al., 2019, 2020). Consequently, it would be scientifically rewarding to further explore the African antitrypanosomal compounds.β-sitosterol is a phytosterol with a chemical structure similar to cholesterol (Figure 1), but can only be synthesized by plants (Bouic et al., 1999). Several pharmacological activities of the compound such as antioxidant, anticancer, and anti-inflammatory among others have been documented (Paniagua-Pérez et al., 2005). More importantly, the anti-leishmanial efficacy of the compound was reported in addition to *in vitro* antitrypanosomal activity against *T. brucei brucei* (Hoet et al., 2007). Although the mechanism of action of β-sitosterol in parasitic infection is not fully understood, the compound has been proposed to potentially disrupt the integrity of the parasite’s lipid bilayer, inhibit some enzyme activities, modulate host immune response, alteration of sterol metabolism and induction of apoptosis in some parasites (Pramanik et al., 2020; David et al., 2021; Kasirzadeh et al., 2021; Elawad et al., 2023; Shokry et al., 2023). Interestingly, our recent investigations employing *in vitro* kinetic analysis and molecular dynamic simulation studies proved the efficacy of the compound against *T. congolense* sialidase and phospholipase A_2_ (Aminu et al., 2023). Considering the wide activity spectrum of the compound and in a bid to further investigate the possible therapeutic effect of β-sitosterol on the trypanosomal infection, we performed an *in vivo* study to investigate the effect of β-sitosterol on *T. congolense*-induced pathological damages and the effect of the compound on the expression pattern of the parasite trans-sialidase gene variants. The information generated can improve the current strategies employed in searching for newer chemotherapeutic agents against *Nagana*.

## 2. Materials and methods

### 2.1. Chemicals and reagents

β-sitosterol, fetuin, and N-acetylneuraminic acid were purchased from Sigma Chemical Company (Saint Louis, MO 63103, United States). Assay kits for serum biomarkers (alanine aminotransferases (ALT), aspartate aminotransferases (AST), creatinine, and urea) were purchased from LABKIT (Chemelex, S.A., Barcelona) while RNeasyprotects animal blood and Maxima H minus Reverse Transcriptase (RT) enzyme kits were purchased from Qiagen V. N Company, Hilden, Germany, and Thermo Fisher Scientific Company, United States, respectively. Sodium periodate and thiobarbituric acid (TBA) were obtained from KEM light laboratories PVT Ltd. India. The standard drug, diminazene aceturate (D.A) used in the study was produced by HebelKexing Pharmaceutical Company Limited, China but purchased from a local veterinary clinic.

### 2.2. Source of trypanosome and experimental animals

The savannah strain of *T. congolense* used in this study was obtained from the stabilates at the National Institute for Trypanosomiasis and Onchocerciasis Research (NITOR), Kaduna-Nigeria. The parasite was validated using PCR with species-specific ITS-1 primers yielding a band size of 640 base pairs (bp). It was allowed to grow to a peak parasitemia (in donor rats) of about 10^9^ parasite/mL of blood. On the other hand, forty-two (42) apparently healthy Wistar rats (150–200 g) were purchased from the Department of Pharmacology and Therapeutics, Ahmadu Bello University, Zaria, and were acclimatized for 1 week before the commencement of the studies. The animals were housed in plastic cages and provided with commercial rat chow (ECWA Feeds, Jos, Nigeria) and water *ad libitum* (maintained at room temperature of 25°Cwith 12 h light and dark cycle). The animal’s handling was according to the guidelines of Good Laboratory Practice (GLP) regulations of the World Health Organization while the study was conducted and reported according to the ARRIVE guidelines.^1^ Ethical approval with the number ABUCAUC/2020/44 was obtained from the Ahmadu Bello University ethical committee before initiating the studies.

### 2.3. Evaluation of the *in vivo* antitrypanosomal efficacy of β-sitosterol

After becoming accustomed to the environment, the animals were randomly grouped into six groups containing seven rats each as follows; Normal Control (NC), Infected Control (IC) animals, Infected treated with an intraperitoneal injection of 3.5 mg/kg BW diminazene aceturate (ITDA), Infected but orally treated with 15 (IT15BS) and 30 mg/kg BW (IT30BS) β-sitosterol as well as an uninfected group but orally treated with 30 mg/kg BW (UT30BS) of the β-sitosterol. For the parasite inoculation, the blood from the infected donor rat was collected and diluted with cold physiological saline to make up for the required inoculum of 10^4^ parasite/mL. About 0.4 mL/100 g of the inoculum was used to infect the experimental animals and the establishment of parasites in their bloodstream was monitored. The appearance of the parasite on the 4th-day post-infection (pi)initiated the daily treatment with respective dosages of β-sitosterol and diminazene aceturate which proceeded for 14 days. In the infected animals, daily parasitemia was monitored using the rapid matching counting method (Herbert and Lumsden, 1976; Aminu et al., 2022) while the pre-and post-infection packed cell volumes (PCV) of the experimental animals were determined using the micro-hematocrit method.

### 2.4. Blood and organ sample collection

At the end of the experiment, the animals’ weights were ascertained and then, were humanely sacrificed under mild anesthesia. Blood samples were collected in both plain and EDTA containers *via* cardiac puncture. Serum was collected from the blood contained in the plain bottles by centrifuging at 3000 × g for 15 min and was stored at 2°C before further analysis. Immediately, RNALater was added to the EDTA-containing blood (1:4) and was stored at -20°C to preserve the total RNA in the samples whereas the liver, and kidney of animals were washed with normal saline, blotted with filter paper, and then weighed in order to determine their relative organ weight using the formula;


$$\mathrm{Relative}\;\mathrm{organ}\;\mathrm{weight}=\frac{\mathrm{Absolute}\;\mathrm{organ}\;\mathrm{weight}\;\left(g\right)}{\begin{array}{l} \mathrm{Live}\;\mathrm{weight}\;\mathrm{of}\;\mathrm{animal}\;\mathrm{on}\;\\ \mathrm{the}\;\mathrm{day}\;\mathrm{of}\;\mathrm{sacrifice} \end{array}}\;\times 100$$

For histopathology, the organs were stored in 10% formalin and stored at 25 ± 2°C prior to analysis.

### 2.5. Determination of biochemical parameters and free serum sialic acid

The serum activities of aspartate and alanine aminotransferase (AST and ALT) in addition to serum urea and creatinine were determined using LABKIT reagents according to the manufacturer’s protocol. Likewise, free serum sialic acid was determined according to the thiobarbituric acid (TBA) method (Aminoff, 1961; Aminu et al., 2017a), where 100 μL of 25 mM periodate solution was added to serum (100 μL), mixed, and allowed to stand at 37°C for 30 min before the addition of 200 μL of sodium arsenate (2%). The mixture was capped and mixed thoroughly. Thereafter, 2 mL of 0.1 M TBA was added to the mixture and heated at 80°C for 8 min. The mixture was cooled for 5 min before the addition of a 2.5 mL acid-butanol reagent. The absorbance of the butanol layer was measured at 549 nm after centrifuging at 3000 rpm for 5 min. The sialic acid concentration of the samples was determined from a sialic acid standard curve.

### 2.6. Histopathology of the kidney

The histopathology of the Kidney was conducted by differential staining using hematoxylin and eosin dyes as previously described (Aminu et al., 2022). Briefly, the kidney contained in 10% formalin was dehydrated, cleared, and infiltrated using graded ethanol, xylene, and molten paraffin wax. The kidney was cut into micro-sections and applied on slides which were later stained using hematoxylin and eosin. Thereafter, the slides were viewed by trained personnel who had no idea of the experimental procedure, and the level of kidney damage was quantitatively graded. Tissues without any damage (0%) or with slight damage (1–20%) were scored as <2. Tissues with mild damage (40%) were graded as 2 < 5, while those with moderate damage (60%) were graded as 5 < 25. Tissues with severe damage (> 60%) were graded as >25.

### 2.7. Isolation, purification and quantification of *T. congolense* RNA

Total RNA was isolated and purified using RNeasy protect animal blood kits (Eppendorf, Germany) following the manufacturer’s instructions. Briefly, blood was centrifuged and washed with water before suspending in re-suspension buffer and digested with proteinase K in binding buffer followed by the addition of molecular grade ethanol. The samples were centrifuged in RNeasy spin columns and DNases were added. Subsequently, the total RNA was washed with buffer to generate the pure RNA which was eluted with an elution buffer. The eluted RNA was chilled immediately on ice and quantified using a Nanodrop spectrophotometer and its purity was determined.

### 2.8. Synthesis of complementary DNA (cDNA)

Complementary DNA (cDNA) was synthesized using maxima H minus reverse transcriptase (Thermo Scientific, Hamburg, Germany) strictly following the manufacturer’s instructions as previously described (Aminu et al., 2022). For the synthesis, the kit components were initially thawed, mixed, centrifuged, and then chilled on ice before the addition of the template RNA (100 ng/mL). Thereafter, 1 μL of random hexamer primers (5′ – d (NNNNNN) –3′; N = G, A, T or C), 1 μL of dNTPs and nuclease-free water was added to make up for the required volume. The mixture was gently mixed, centrifuged, and incubated at 37°C for 2 min. Additionally, 5 μL of Maxima cDNA H minus Synthesis Master Mix (5X) was added to make the entire reaction volume of 19.5 μL, and the mixture was centrifuged and incubated at 25°C for 10 min. Further incubation at 50°C for 30 min was done while the reaction was stopped by heating at 85°C for 5 min. The generated cDNA was used in the Real Time Quantitative Polymerase Chain Reaction (RT-Q PCR).

### 2.9. Real-time quantitative PCR and absolute quantification of *T. congolense* trans-sialidase gene variants

The qPCR was performed in a 10 μL volume containing 0.2 ng cDNA, 1 μM of each primer, 0.1 μM of each FAM-labeled probe, and 2x Taqman Fast Universal PCR Master Mix (Applied Biosystems). The reaction was performed in a Step-One Plus Real-Time PCR System (Applied Biosystems, United States). The following cycling conditions were employed: 95°C for 30 s, 60°C for 45 s, and 72°C for 60 s. The experiment was done in triplicates. The primers and probe sequences (Chechet, 2015) used in the qPCR reaction are as follows: *TconTS1* (Fwd 5^′^-CTGACGATGGAAAGTCATGG-3^′^; Rev. 5^′^-ATCATACGGTAGCCCTGTCC-3^′^; Probe FAM 5^′^-TCCGAGGCTGCCCTCACTG-3^’^ BHQ1), *TconTS2* (Fwd 5^′^-GCCATAACTGTGGAGGGAGT-3^′^; Rev. 5^′^-AATCTGTCCAACAAGCCACA-3^′^; Probe FAM 5^′^-ATCGCGACCGAATGCGACTG-3^′^ BHQ1), *TconTS3* (Fwd 5^′^-TTCATCAAGTCGCACTCACA-3^′^; Rev. 5^′^-GGATGCCCAACAAAGAAGTT-3^′^; Probe FAM 5^′^-CGCCCGCAACCTTCGTATCC-3^′^ BHQ1), *TconTS4* (Fwd 5^′^-TCGCCGAAAGCAACTATATG-3^′^; Rev. 5^′^-AACCCGTCAGCAGCTCTTAT-3^′^; Probe FAM 5^′^-TTGTCCCTCAACCGGGAGGC-3^′^ BHQ1).

### 2.10. Statistical analysis

Data obtained were expressed as mean ± standard deviation and were analyzed using GraphPad Prism version 5. Except for PCV, the data generated were analyzed using one-way analysis of variance (ANOVA) followed by Dunnet (for parasitemia; IC served as a control) and/or Tukey’s-HSD multiple *post hoc* tests in order to test for significance. For the PCV, a paired-sample t-test was conducted where differences in pre and post-infection values were determined. In all cases, *p* values less than 0.05 were considered statistically different. To determine the relationship between the free serum sialic acid and the *T. congolense* trans-sialidase gene variants, a Pearson correlation was conducted and significance at *p* < 0.01 was considered.

## 3. Results

**Figure 2:**
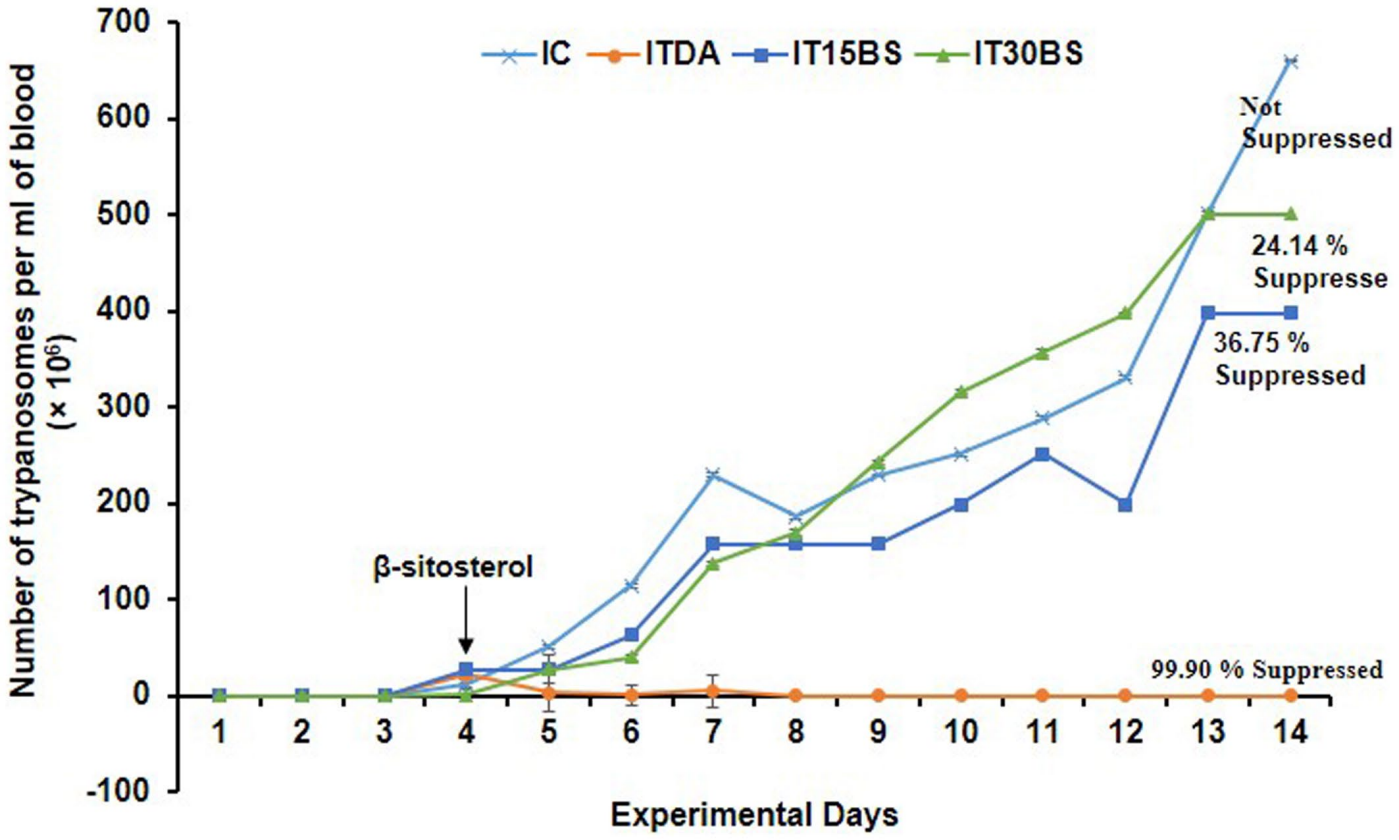
Therapeutic efficacy of oral administration of β-sitosterol on bloodstream *T. congolense* parasitemia. For parasitemia determination, the data are presented as mean ± standard deviation of seven rats. Dunnett posthoc test was used to analyze the data following one-way ANOVA. IC was used as a control. IC, infected control; IT15ΒS, infected +15 mg/kg BW β-sitosterol, and IT30BS, infected +30 mg/kg BW β-sitosterol; ITDA, infected +3.5 mg/kg BW diminazene aceturate.

On day 4 p.i, *T. congolense* appeared in the infected groups indicating successful inoculation and hence, marked the immediate initiation of β-sitosterol treatment (Figure 2). Observably, a progressive increase in the parasite load was observed in the infected control (IC) group throughout the experimental period except on day 8 p.i where a fluctuation was recorded (Figure 2). A similar increase was observed in β-sitosterol treated groups with the infected but treated with 30 mg/kg BW (IT30BS) group exhibiting significant (*p* < 0.05) parasitemia between day 10–12 pi but it was significantly lowered by day 14 pi compared to the IC group. Although there was an increase in parasite load in the infected-treated with 15 mg/kg BW (IT15BS) group, the value was significantly (*p* < 0.05) lower than the IC group throughout the experimental period. Noticeably, the parasite completely disappeared in the ITDA group the same day treatment was initiated (Figure 2).

In order to determine the therapeutic potential of β-sitosterol on anemia, the packed cell volume (PCV) of the animals was investigated. The final PCV in the IC group significantly (*p* < 0.05) dropped in relation to their baseline packed cell volume (PCV) value (Table 1). Treatment with β-sitosterol at both 15 and 30 mg/kg BW dosages significantly (*p* < 0.05) reversed the PCV value of the infected animals. Further analysis of the anemia-ameliorative effect of β-sitosterol showed a % change in PCV of −28.82 and − 33.69% in the IT15BS and IT30BS groups respectively, which were relatively lower than −76.55% observed in the IC group (Table 1).

**Table 1:** Anemia-ameliorative effects of oral administration of β-sitosterol in *T. congolense*-infected animals.

Group/Treatment	Pre-infection PCV (%)	Post-infection PCV (%)	change in PCV (%)
NC	38.00 ± 2.94^a^	47.25 ± 8.62^a^	+19.58
IC	53.67 ± 8.60^b^	30.40 ± 2.30^a^	−76.55
UT30BS	45.60 ± 2.50^a^	46.80 ± 4.66^a^	+2.56
IT15BS	54.75 ± 0.50^b^	38.67 ± 4.04^a^	−28.82
IT30BS	48.57 ± 6.82^b^	40.00 ± 1.41^a^	−33.69
ITDA	43.20 ± 2.17^a^	53.25 ± 9.43^a^	+18.87

All data are presented as mean ± standard deviation of seven rats. Data were analyzed using Paired sample t-test. Values with different letters (a, b) are considered statistically significant within groups, at *p* < 0.05. NC, Normal control; IC, Infected control; UT30BS, Un-infected + 30 mg/kg BW β-sitosterol; IT15ΒS, Infected + 15 mg/kg BW β-sitosterol; IT30BS, Infected + 30 mg/kg BW β-sitosterol; ITDA, Infected + 3.5 mg/kg BW diminazine aceturate; PCV, Packed cell volume.

**Figure 3:**
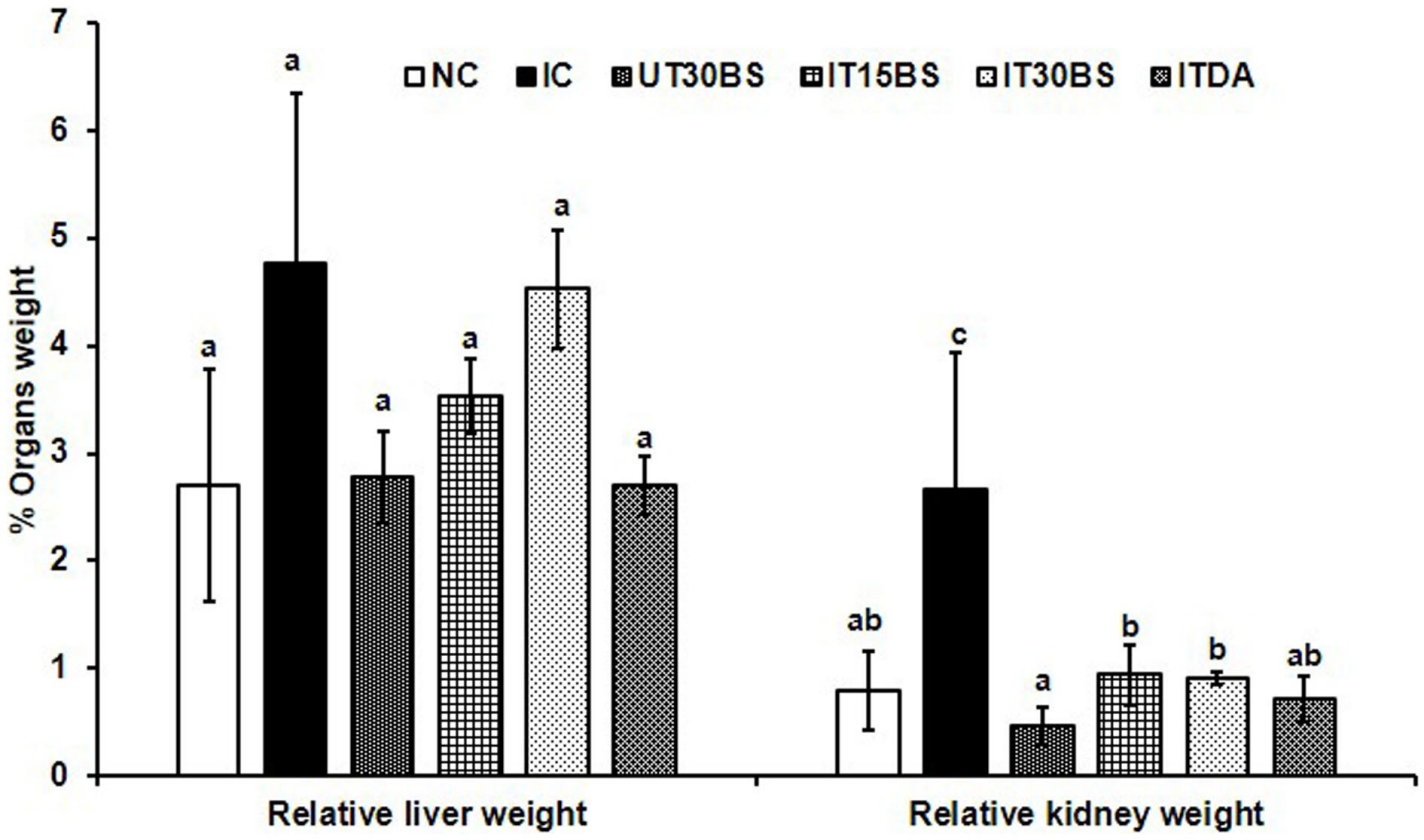
Therapeutic efficacy of oral administration of β-sitosterol on the relative liver and kidney weights in *T. congolense*-infected animals. Values with different letters between groups are considered statistically significant at *p*< 0.05. NC, Normal control; IC, infected control; UT30BS, un-infected +30 mg/kg BW β-sitosterol; IT15ΒS, infected +15 mg/kg BW β-sitosterol and IT30BS, infected +30 mg/kg BW β-sitosterol; ITDA, infected +3.5 mg/kg BW diminazene aceturate.

Trypanosomal infection is accompanied by hepatic and renal damage, and this was prominent in the study. Infection with *T. congolense* in the IC group was accompanied by significant (*p* < 0.05) elevation in some biochemical indices of hepatic and renal damage compared with the normal control group (Table 2). The hepatic biomarkers particularly the AST were not affected by the treatment while the compound at both dosages significantly (*p* < 0.05) reduce the renal damage induced by the parasite in the infected animals (Table 2). Furthermore, investigating the relative hepatic and renal weights of the animals revealed a non-significant (*p* > 0.05) increase in relative liver weight in the IC group compared with the normal control group (Figure 3). Treatment with both dosages of the β-sitosterol showed a non-significant (*p* > 0.05) increase in the relative liver weight (Figure 3). With respect to the kidney, a significant (*p* < 0.05) increase in the weight of kidneys in IC animals was observed compared with the NC group (Figure 3). However, the kidney enlargement was reduced following β-sitosterol administration at both dosages, respectively, (Figure 3).

**Table 2:** Therapeutic efficacy of oral administration of β-sitosterol on biochemical parameters in *T. congolense*-infected animals.

Parameters	NC	IC	UT30BS	IT15BS	IT30BS	ITDA
Serum ALT (U/L)	2.33 ± 1.65^a^	2.65 ± 0.58^a^	4.67 ± 1.65^a^	3.89 ± 1.78^a^	36.17 ± 4.95^b^	6.61 ± 3.36^a^
Serum AST (U/L)	1.74 ± 0.83^a^	2.13 ± 0.89^a^	3.20 ± 1.24^a^	3.45 ± 0.02^a^	2.91 ± 1.65^a^	2.91 ± 0.58^a^
Urea (mg/dL)	36.62 ± 1.74^a^	43.44 ± 1.34^b^	33.42 ± 6.28^a^	42.21 ± 2.56^b^	32.92 ± 0.62^a^	50.00 ± 5.58^b^
Creatinine (mg/dL)	0.99 ± 0.07^a^	2.02 ± 0.47^b^	1.09 ± 0.46^a^	4.89 ± 2.03^c^	1.08 ± 0.24^a^	2.44 ± 0.87^bc^

The data are presented as the mean ± standard deviation of seven rats. Datawere analyzed using ONE-WAY ANOVA followed by (Tukey’s multiple rangesposthoc test). Values with different letters between groups are considered statistically significant at *p* < 0.05.AST, Aspartate aminotransferases; ALT, Alanine aminotransferases; NC, Normal control; IC, Infected control; UT30BS, Un-infected + 30 mg/kg BW β-sitosterol; IT15ΒS, Infected + 15 mg/kg BW β-sitosterol; IT30BS, Infected + 30 mg/kg BW β-sitosterol; ITDA, Infected + 3.5 mg/kg BW diminazine aceturate.

**Figure 4:**
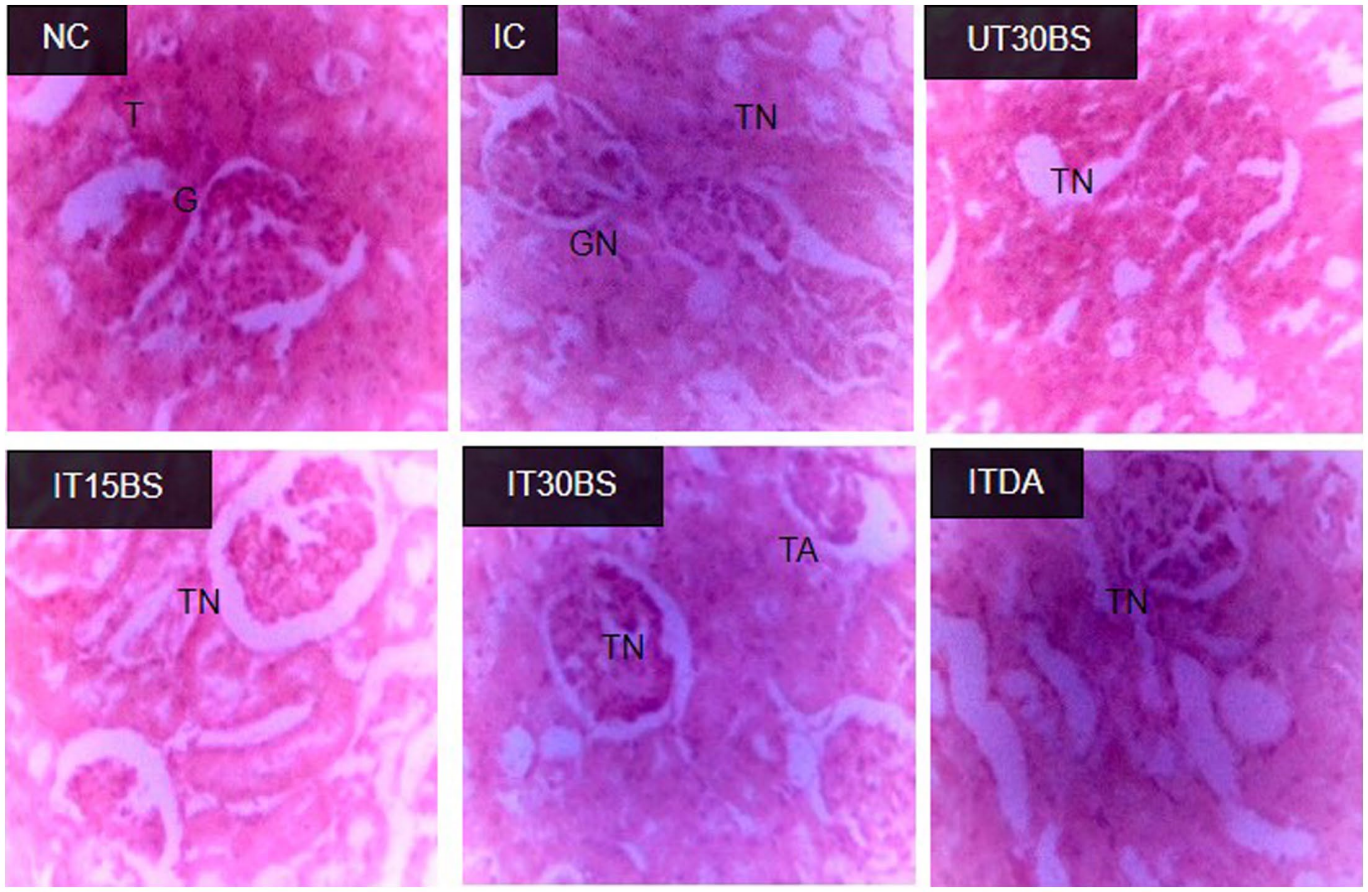
Effect of oral administration of β-sitosterol on the renal histopathological changes observed in *T. congolense-infected* animals. NC, Normal control; IC, infected control; UT30BS, un-infected +30 mg/kg BW β-sitosterol; IT15ΒS, infected +15 mg/kg BW β-sitosterol and IT30BS, infected +30 mg/kg BW β-sitosterol; ITDA, infected +3.5 mg/kg BW diminazene aceturate. T, Normal Tubules; G, Normal Glomerulus; GN, Glomerular Necrosis; TA, Tubular Adhesion; TN, Tubular Necrosis.

**Figure 5:**
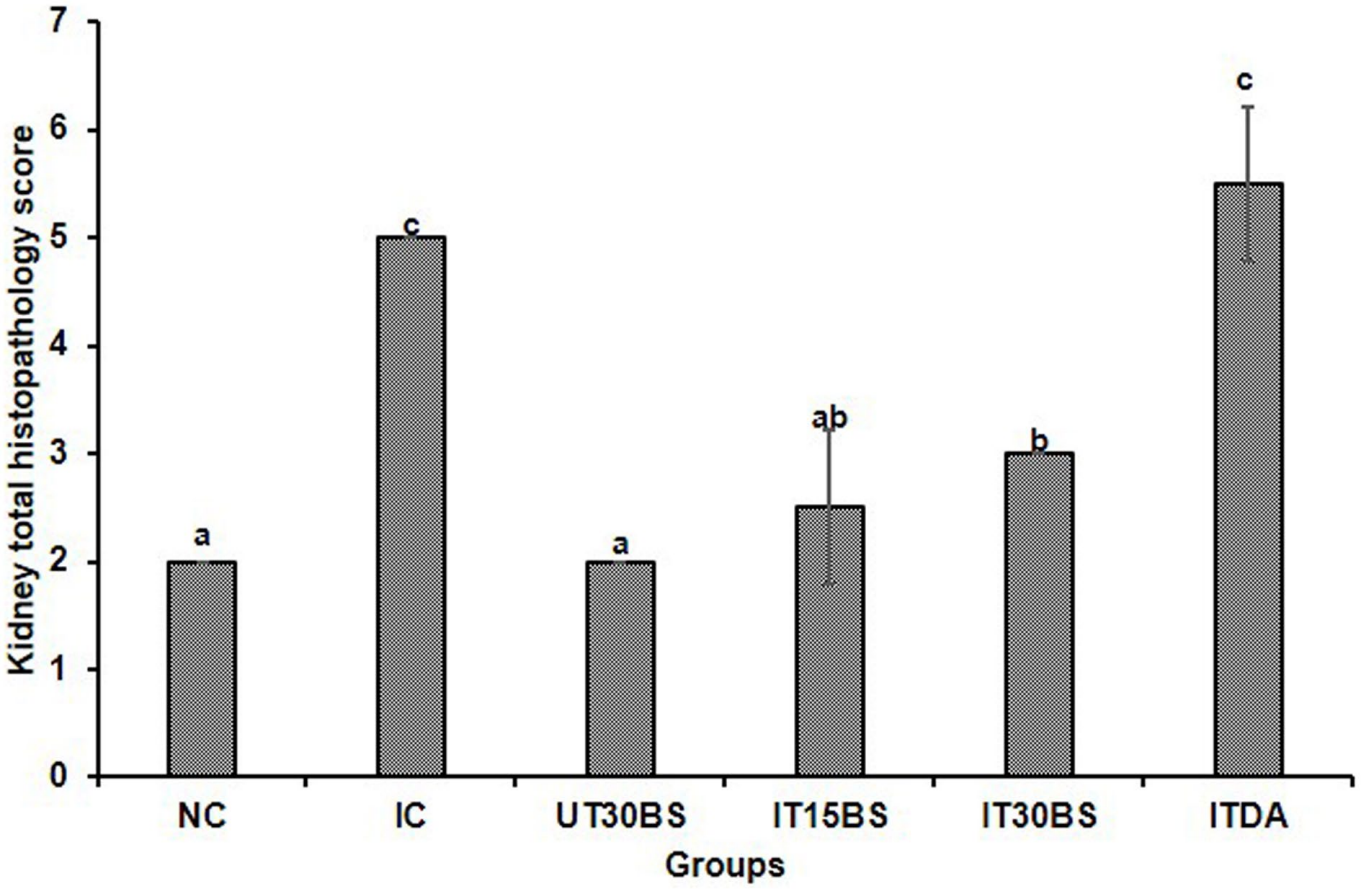
The overall quantitative kidney histopathological scores of *T. congolense-infected* animals following oral administration of β-sitosterol. The data are presented as the mean ± standard deviation of three rats. Data were analyzed using one-way ANOVA followed by (Tukey’s multiple ranges posthoc test). Values with different letters between groups are considered statistically significant at *p* < 0.05. NC, Normal control; IC, Infected control, UT30BS, Un-infected +30 mg/kg BW β-sitosterol; IT15ΒS, Infected +15 mg/kg BW β-sitosterol; IT30BS, Infected +30 mg/kg BW β-sitosterol; ITDA, Infected +3.5 mg/kg BW diminazine aceturate.

In order to corroborate the effect of the compound on the kidney, the histopathology of the kidney was conducted. Observably, glomerular necrosis (GN) and tubular necrosis (TN) were observed in the IC group (Figure 4). In the treatment and diminazene aceturate-treated groups, tubular necrosis (but not glomerular necrosis) was observed (Figure 4). Also, the quantitative investigation of the total damage revealed a significant (*p* < 0.05) total damage score in the IC group compared to the normal control group (Figure 5). Treatment with 15 mg/kg and 30 mg/kg BW β-sitosterol significantly (*p* < 0.05) reduced the kidney total damage score and hence could suggest the pathologic damages induced by the parasite in the kidney were ameliorated by the compound (Figure 5).

**Figure 6:**
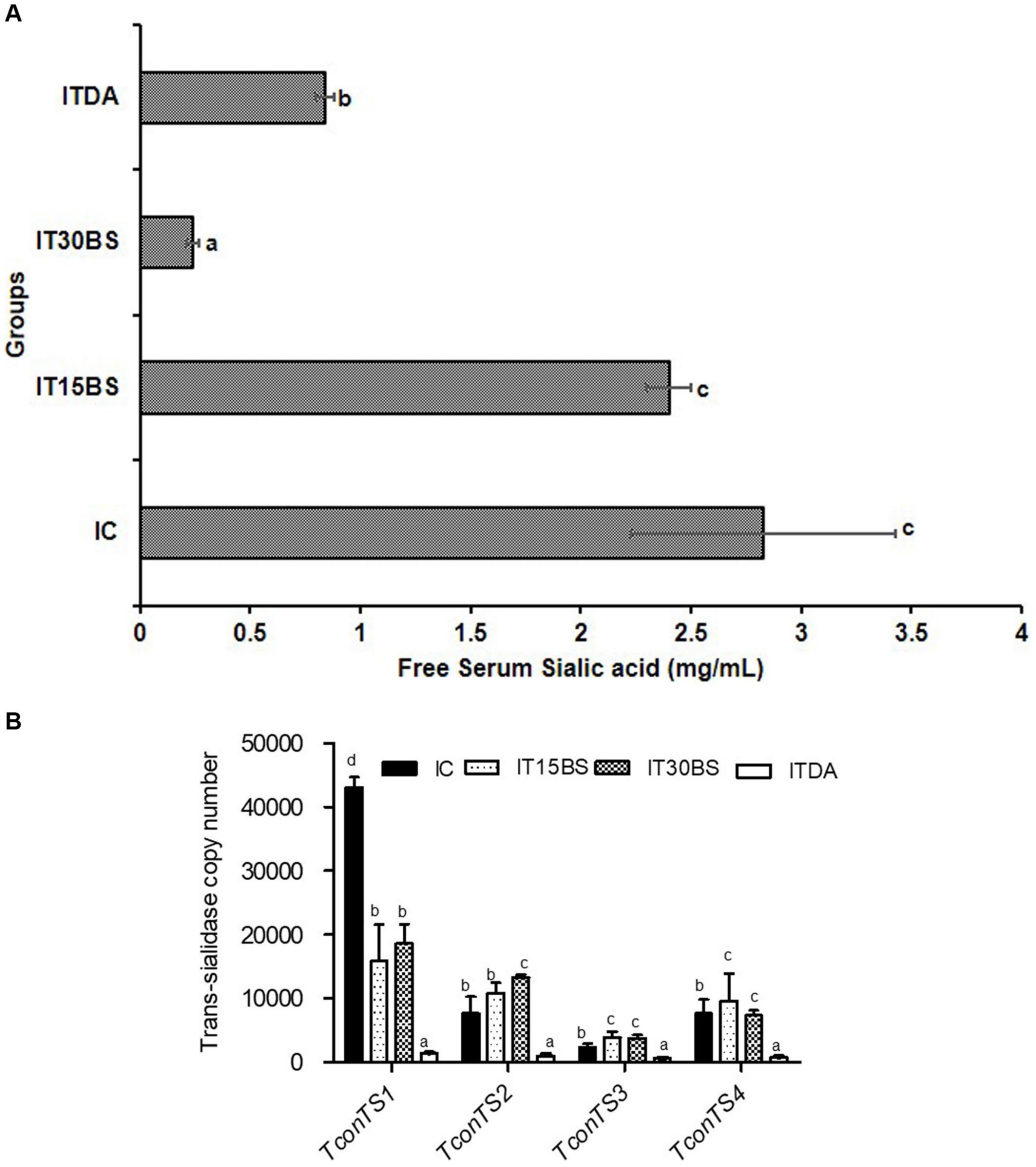
The free serum sialic acid in *T. congolense*-infected animals **(A)** and mRNA expression profile of bloodstream *T. congolense* trans-sialidase gene variants following oral administration of β-sitosterol to *T. congolense*infected animals **(B)**. The data are presented as the mean ± standard deviation of seven rats. Data were analyzed using one-way ANOVA followed by (Tukey’s multiple ranges posthoc test). Values with different letters between groups are considered statistically significant at *p* < 0.05. NC, Normal control; IC, Infected control; UT30BS, Un-infected +30 mg/kg BW β-sitosterol; IT15ΒS, Infected +15 mg/kg BW β-sitosterol; IT30BS, Infected +30 mg/kg BW β-sitosterol; ITDA, Infected +3.5 mg/kg BW diminazine aceturate.

*T. congolense* infection is accompanied by the release of sialic acid and the parasite is known to scavenge the sialic acid using its enzyme machinery (particularly, the (trans)-sialidase enzyme). In this study, the amount of free serum sialic acid and the expression of *T. congolense* trans-sialidase gene variants were investigated in the infected animals (Figure 6A). A significant (*p* < 0.05) elevation in the free serum sialic acid was observed in the infected control (IC) group (Figure 6A). However, the level of the sialic acid was significantly reduced in animals treated with 30 mg/kg BW β-sitosterol (Figure 6A). With respect to the mRNA level of the trans-sialidase variants, the observed overexpression of *TconTS1* in the IC group was significantly (*p* < 0.05) reduced upon treatment with 15 and 30 mg/kg BW β-sitosterol respectively, while the compound led to a significant (*p* < 0.05) increase in the expression of *TconTS2* variant in the IT30BS group (Figure 6B). In the same vein, expressions of *TconTS3* and *TconTS4* in the IT15BS and IT30BS were significant (*p* < 0.05) increased compared with the IC group, indicating that the compound has no effect on the variants (Figure 6B). Pearson correlation between free serum sialic acid with the *TconTS1* and *TconTS2* gene variants showed strong negative correlations (*p* < 0.05) in the 30 mg/kg BW β-sitosterol groups (Table 3). Similarly, a negative correlation (*p* > 0.05) was observed between free serum sialic acid with the *TconTS3* and *TconTS4, respectively* (Table 3). Although the correlations were also negative between the biomolecule and the *TconTS* gene variants in the 15 mg/kg BW β-sitosterol treated groups, the values were non-significant (*p* > 0.05) (Table 3). In the IC group, a strong negative correlation (*p* < 0.05) was observed between free serum sialic acid with *TconTS1* and positive correlations were observed between free serum sialic acid with the other gene variants (Table 3). In contrast, positive correlations (*p* < 0.05) were observed between free serum sialic acids and *TconTS1*, *TconTS3*, and *TconTS4* gene variants in the ITDA group, although the correlation was negative with respect to *TconTS2* (Table 3).

**Table 3:** Relationship between free serum sialic acid with *T. congolense* trans-sialidase gene variants in *T. congolense*-infected animals treated with β-sitosterol.

Groups	Correlation (2-tailed Significant value)			
	*TconTS1*	*TconTS2*	*TconTS3*	*TconTS4*
IC	−1.000** (0.01)	0.979 (0.132)	1.000** (0.01)	0.996 (0.06)
IT15BS	−0.287 (0.185)	−0.442 (0.709)	−0.002 (0.998)	0.077 (0.923)
IT30BS	−1.000** (0.01)	−1.000** (0.01)	−0.587 (0.413)	−0.528 (0.472)
ITDA	1.000** (0.01)	−1.000** (0.01)	1.000** (0.01)	1.000** (0.011)

Pearson correlation was used to determine the existing relationship between the parameters. Values are the correlation coefficient (r) of two continuous variables. IC, Infected control; IT15ΒS, Infected + 15 mg/kg BW β-sitosterol; IT30ΒS, Infected + 30 mg/kg BW β-sitosterol; ITDA, Infected + 3.5 mg/kg BW diminazene aceturate; *TconTS*, *T. congolense* trans-sialidase; **, statistical difference at *p* less than 0.01.

## 4. Discussion

Even with the renewed efforts by the scientific communities to control AAT, the disease has continued to pose a great constraint to livestock production in sub-Saharan Africa (Meyer et al., 2016; Habeeb et al., 2021) mainly because the available trypanocides are faced with major drawbacks (Assefa and Shibeshi, 2018). In light of this, the present study demonstrated the therapeutic benefits of β-sitosterol on *T. congolense* proliferation, anemia amelioration, kidney damage, and the *TconTS1* gene expression.

The sustained increase in *T. congolense* in the IC group throughout the experimental period is a known phenomenon in trypanosomiasis and seemed to be responsible for the major pathologies observed in the infected host (Mugnier et al., 2015). Although treatment with 15 mg/kg BW β-sitosterol manifested a positive effect immediately, the 30 mg/kg BW β-sitosterol becomes effective mainly at the last 2 days of the study. In both cases, the compound appeared to be trypanostatic with parasite suppression below 40%. The low efficacy observed could be related to the high IC_50_ of the compound recorded against *T. brucei brucei* (Hoet et al., 2007). The inability of the compound to clear parasites could be due to their low bioavailability as only a small amount of administered phytosterols are known to be absorbed and reach the systemic circulation and their concentration is usually 200 times lower than the concentration of cholesterol under normal nutrition (Ostlund, 2007; Ras et al., 2013). Conversely, β-sitosterol was known to exhibit anthelmintic and anti-leishmanial activities (Vijaya and Yadav, 2014; Majid-Shah et al., 2019), the latter disease type has a similar etiology to trypanosomiasis (Lawyer and Perkins, 2000).

Even with the low efficacy of β-sitosterol towards clearing the *T. congolense*, the parasite-induced anemia was reverted following treatment with the respective dosages. This could be a remarkable observation since anemia has been reported to be a major symptom associated with the disease (Balogun et al., 2014). Most often, death associated with trypanosomiasis was positively correlated with anemia development (Igbokwe, 2018). Although the treatment reversed anemia, the compound does not maintain the PCV values of the infected animals to their initial status. The inability of the compound to maintain the normal PCV levels of the infected animals could occur due to its inability to completely clear the parasite since a negative correlation between parasitemia and PCV is a known phenomenon during infection (Fidelis-Junior et al., 2016).

In addition to anemia, organ degeneration has been known as another devastating pathologic feature associated with trypanosomiasis (Umar et al., 2009). Observably, damages to the liver and kidney of the *T. congolense*-infected animals were prominent in our study since an increase in serum hepatic and renal biomarkers was recorded. Organ damage has been associated with oxidative stress imposed by the parasite (Ibrahim et al., 2016). Even with β-sitosterol treatment, the serum aminotransferases were unaffected while the serum urea and creatinine were ameliorated.

Although we have previously reported the anemia-ameliorating effect of β-sitosterol by targeting sialidase and phospholipase A_2_ (Aminu et al., 2023), the observed compound’s ability to prevent renal hypertrophy could also be considered as an additional factor in reducing anemia. This is because, the concept of renal hypertrophy has been reported as a factor contributing to anemia development during trypanosomiasis (Stijlemans et al., 2018). The 30 mg/kg BW of the compound showed more efficacy than the lower dosage (15 mg/kg BW). Previous administration of a relatively higher dosage of β-sitosterol (20 mg/kg BW) to rats induced with nephrotoxicity showed significant positive changes in renal biochemical and histopathological changes (Sharmila et al., 2016). This could means that, more positive effect might be observed by increasing the dosage. Hence, this could be an important finding as the ability of the compound to maintain renal integrity could be explored in designing promising drug agents.

In addition to the effect of the compound in ameliorating renal hypertrophy, the observed effect of 30 mg/kg BW β-sitosterol towards preventing the release of free serum sialic acid is also an important finding since the cleavage of the sialic acid by trans-sialidases has been implicated in anemia development (Guegan et al., 2013). Supposedly, compounds possessing trypanostatic and anemia-ameliorating potentials could as well, prevent the release of free sialic acid as observed with β-sitosterol (Ajakaiye et al., 2013). Considering the importance of the sialic acid to the parasite, it would be worthwhile to investigate and correlate the expression level of *TconTS* and the sialic acid in the infected animals (Coustou et al., 2012). To achieve this, absolute quantification of the variants was conducted. This allows an actual measurement of the absolute amount of the variants which was used to correlate with the amount of sialic acid in the animals. Of all the gene variants, it was only the expression of *TconTS1* and not *TconTS2*, *TconTS3,* and *TconTS4* that was reduced after treatment with β-sitosterol. The *TconTS1* with *TconTS2* were reported to have high trans-sialidase activity and hence, the effect of the compound on the *TconTS1* could suggest decreased trans-sialylation of the parasite surface molecules (Gbem et al., 2013) while the increase in the expression of *TconTS3* and *TconTS4* could suggest enhanced sialic acid hydrolysis (Gbem et al., 2013; Balogun et al., 2014). However, to better understand the significance of these findings, it would be highly appealing to perform relative quantification. With regards to the study objectives, correlating the free serum sialic acid and the variants was performed and the observed positive correlation, especially in IC and ITDA groups, between free serum sialic acid with the*TconTS3* and *TconTS4* suggests reduced cleavage of sialic acid from host erythrocytes, decreased erythrophagocytosis, and improved survival (Coustou et al., 2012) while the observed negative correlations following β-sitosterol treatment may indicate that the decrease in the expression of *TconTS1* gene variant could not be the most important contributor to the anemia-ameliorative effects of the compound.

## 5. Conclusion

In conclusion, it was observed that β-sitosterol possessed *in vivo* therapeutic efficacy on bloodstream *T. congolense* but could not completely clear the parasites from the blood of the infected rats. At the same time, the compound was able to ameliorate anemia and renal damage induced by the parasite as clearly supported by the histopathological investigation. Nonetheless, the compound was also reported to prevent the parasite-induced release of free serum sialic acid and reduce the expression of the *TconTS1* gene variant. In order to elucidate more targets, the compound should be further studied by targeting other factors enhancing the progression of the disease. Also, there is a need for structural modification of the compound to improve its antitrypanosomal efficacy, particularly in clearing the parasite.

## Data availability statement

The original contributions presented in the study are included in the article/supplementary material, further inquiries can be directed to the corresponding authors.

## Ethics statement

The animal study was approved by Ahmadu Bello University ethical committee. The study was conducted in accordance with the local legislation and institutional requirements.

## Author contributions

SA: Conceptualization, Formal analysis, Investigation, Methodology, Resources, Writing – original draft. GC: Investigation, Methodology, Resources, Writing – review & editing. SSA: Formal analysis, Funding acquisition, Investigation, Resources, Writing – review & editing. MS: Formal analysis, Funding acquisition, Investigation, Resources, Writing – review & editing. RD: Formal analysis, Funding acquisition, Investigation, Resources, Writing – review & editing. MBS: Formal analysis, Funding acquisition, Investigation, Resources, Writing – review & editing. EO: Formal analysis, Funding acquisition, Investigation, Methodology, Resources, Supervision, Validation, Writing – review & editing. MAI: Conceptualization, Formal analysis, Funding acquisition, Investigation, Methodology, Project administration, Resources, Supervision, Validation, Writing – review & editing.

## Footnotes

^1^ https://arriveguidelines.org/
